# Immediate Compression of the Common Iliac

**Published:** 1874-01

**Authors:** Frank Woodbury

**Affiliations:** Resident Physician at the Pennsylvania Hospital


### Immediate Compression of the Common Iliac Artery for the Prevention of Hemorrhage during Operations upon the Lower Extremity, particularly in Reference to its Usefulness in Hip-joirt Amputations.

By Frank Woodbury, M,D., Resident Physician at the Pennsylvania Hospital.

In the performance of an operation involving section of the femoral artery in its upper portion, the absolute control of the circulation through the vessel, for a few moments, is of inexpressible importance, and may decide the success or failure of the operation, the life or death of the patient. The difficulty of doing this effectually constitutes the great immediate danger in the operation of amputation at the hip-joint. To meet this requirement surgeons have adopted aortic compression by Dupuytren's or Pancoast's abdominal tourniquet. That these have proved valuable adjuncts, and that their use has been attended by success, cannot be denied; nor can the fact that they fail to accomplish the result as effectually and with the degree of certainty that are so eminently desirable in such cases; nor are they entirely free from the suspicion that the amount of pressure required may be prejudicial to some of the viscera. When the abdominal tourniquet is applied so as to stop the circulation in the inferior extremities, everything between the pad of the tourniquet and the vertebral bodies must sustain an undeniably abnormal if not pathogenetic degree of pressure. What influence this may exert in the production of the shock after the operation by compression of the solar plexus, or what may be its secondary effects upon the mesentery, intestine, or great vessels themselves (including the vena cava), has not been, until now, taken into consideration as weighed against the great emergency which called the abdominal tourniquet into being; but where the object can be more quickly, certainly, and effectively attained by a simple manual procedure, these accidents command respect, and, where it is possible, should certainly be avoided.

In the examination of a case of nephritic colic, in which a stone was supposed to exist in the renal pelvis, or in the ureter, the writer found that while exploring the abdominal cavity, with the right hand and part of forearm in the rectum, he was able to observe that no stone of size existed in the parts indicated, and was struck by the novel sensation produced by the pulsation of the commom iliac arteries and abdominal aorta immediately under the fingers. The idea occurred that the abdominal aorta and iliac vessels might be compressed and controlled with as much certainty and almost as readily as the radial artery. The value of this where the circulation is required to be checked for a brief period, in either or both lower extremities, is sufficiently evident, but is enhanced by the fact that the compresion is made by a sensitive fleshy pad directly upon the vessel, and the venous trunk is avoided. As the patient is always anæsthetized in large operations, compression need not be instituted until the surgeon is ready to commence the external incision; it can be cautiously removed and instantly reapplied if necessary, and is immediately removed when the vessel is secured, thus maintaining the pressure during the *minimum* of time. The bowel should be evacuated by a large warm-water injection previous to the operation. In controlling the right common iliac artery the right hand of the assistant will be the most convenient, and the left vessel will be most easily controlled by the left hand. In either case, the hand being anointed (with lard in preference to oil), and the fingers folded into a cone, it is gradually introduced into the rectum with its dorsum to the sacrum until reaching the sigmoid flexure, when the hand may be pronated, and, as the vessels are right under the fingers, the main supply of blood to the limb may thus, in a few moments, be completely controlled; if, in conjunction with this, the plan recommended by Esmarch, of Heidelburg, be practised, the operation of amputation at the hip-joint may be rendered almost as bloodless as some of the operations of minor surgery. Other applications will suggest themselves as the exigency occurs requiring the checking of the circulation in the limb, such as in the operation for femoral aneurism high up, or in operations lower down, where the size of the thigh makes the application of a tourniquet on the femoral difficult if not impossible.

The value of recto-abdominal exploration has been generally acknowledged, and, if in the *eclat* of its first appearance, or the excitement of its reintroduction with new and valuable applications, the operation has been misapplied and even abused by surgeons in their haste to accept such a welcome aid to diagnosis, there also exist cases, as in the diagnosis of the supposed tubal calculus before mentioned, and in the application herein suggested, where it is of undeniable importance. The sphincter muscle of the anus, as in most of the reported cases, regains its tone in a few days, and in tardy cases may be hastened to that result by the daily Faradaic current. Superficial lacerations, fissures of the anus, or hemorrhoids need not occur if a little time be taken in introducing the hand, so that the internal sphincter may have time to yeild; when they do occur they rapidly heal under simple treatment."--*American Jour. Med. Sciences.*

